# Effect of maternal isolated hypothyroxinemia in the first trimester on offspring neurodevelopment: a prospective cohort study

**DOI:** 10.3389/fendo.2026.1734708

**Published:** 2026-02-18

**Authors:** Jing Du, Xiumei Xu, Ning Yuan, Xiaomei Zhang

**Affiliations:** Department of Endocrinology and Metabolism, Peking University International Hospital, Beijing, China

**Keywords:** attention deficit hyperactivity disorder, autism spectrum disorder, hypothyroxinemia, intelligence, offspring, pregnancy

## Abstract

**Objective:**

This study aimed to examine the effect of maternal isolated hypothyroxinemia (IH) during early pregnancy on the neuropsychological development of the offspring.

**Methods:**

This single-center prospective cohort study included 100 mother–child pairs, with 50 pairs in the IH group and an equal number in the euthyroid group. Levels of free thyroxine, thyroid-stimulating hormone, and thyroid peroxidase antibody in maternal serum were measured during the first trimester. Offspring neurodevelopment was evaluated using the Chinese Developmental Scale for Children Aged 0–6 Years (2^nd^ Edition), Conners Parent Symptom Questionnaire, and Social Responsiveness Scale (SRS).

**Results:**

After adjusting for confounding factors, multiple linear regression analysis indicated that maternal IH in the first trimester was significantly associated with a decreased developmental quotient in the offspring (adjusted β = −3.28, 95% CI: −5.09 to −1.47, P < 0.001). The highest association was observed in the language domain (adjusted β = −5.72, 95% CI: −8.00 to −3.43, P < 0.001). Moreover, first-trimester IH exposure was linked to increased scores on the offspring’s impulsive–hyperactive behavior (adjusted β = 0.38, 95% CI: 0.16–0.60, P = 0.001) and hyperactivity index (adjusted β = 0.23, 95% CI: 0.06–0.41, P = 0.011). In addition, the offspring’s social awareness (adjusted β = 1.21, 95% CI: 0.10–2.31, P = 0.035) and social communication (adjusted β = 3.01, 95% CI: 0.50–5.53, P = 0.021) subscale scores as well as the total SRS score (adjusted β = 6.51, 95% CI: 0.33–12.69, P = 0.042) were elevated.

**Conclusion:**

Maternal IH in the first trimester is associated with lower intellectual developmental scores and higher scores on screening scales for symptoms related to attention-deficit/hyperactivity disorder and autism spectrum disorder in the offspring.

## 1 Introduction

The first stage of fetal brain development occurs during the first 20 weeks of gestation, during which most neural development is completed. Thyroid hormones (THs) play a crucial role in this process (1–3). However, fetal thyroid development begins only at 12 weeks of gestation. THs are produced and secreted between 12 and 14 weeks (4, 5). In early pregnancy, almost all THs required for fetal development are derived from the mother (6, 7). Therefore, even mild maternal TH deficiency during early pregnancy can impair fetal brain development and lead to neuropsychiatric disorders in the offspring (8, 9).

A previous study found that free triiodothyronine (FT3) is the primary TH directly involved in fetal neurodevelopment (10). Nonetheless, thyroxine (T4), rather than triiodothyronine T3, is the predominant maternal TH that can pass through the placental barrier into the fetus (11). Fetal brain development relies on T4 in a tissue-specific and preferential manner (12). Once maternal T4 enters the fetal brain via the placenta, it is converted to FT3 by deiodinases and plays a vital role in neurogenesis, neuronal proliferation and migration, dendrite and axon growth, synaptogenesis, and myelination (1, 2, 13). An earlier study identified that maternal FT4 level at 12 weeks of gestation was the strongest predictor of fetal brain development (14). Therefore, maternal FT4 sufficiency during pregnancy, especially in the first half, is crucial for ensuring normal neurodevelopment in the offspring. Normal maternal T3 levels cannot compensate for potential damage caused by insufficient T4 supply (15).

Isolated hypothyroxinemia (IH) during pregnancy is defined as serum FT4 levels below the 2.5^th^ or 5^th^ percentile of the pregnancy-specific reference range, with normal serum thyroid-stimulating hormone (TSH) levels (16). Emerging evidence suggests that maternal IH during pregnancy adversely affects the neurodevelopment of the offspring, potentially resulting in delayed motor and intellectual development (17–19), attention-deficit hyperactivity disorder (ADHD) (20, 21), autism spectrum disorder (ASD) (22, 23), and schizophrenia (24). Nevertheless, current investigations on the link between gestational IH and offspring neurodevelopment remain few and inconclusive. Hence, additional studies are required to explore the effect of maternal IH during pregnancy on the neurodevelopment of the offspring. This prospective study aimed to examine the impacts of maternal IH in the first trimester on offspring neurodevelopmental outcomes. The findings are expected to provide additional evidence for the clinical management of IH during pregnancy and for improving maternal–fetal health.

## 2 Materials and methods

### 2.1 Participants and grouping

Pregnant women and their offspring who registered and delivered at the obstetrics department of Peking University International Hospital from December 2016 to June 2019 were enrolled in this single-center, prospective cohort study.

Inclusion criteria for mothers (1): age 20–40 years (2); Beijing residency >5 years (3); singleton pregnancy (4); received antenatal care and delivered at the hospital. Exclusion criteria for mothers (1): multiple gestation (2); history of thyroid disorders (such as clinical/subclinical hyperthyroidism/hypothyroidism, and thyroid cancer) (3); use of medications that affect thyroid function (levothyroxine, antithyroid drugs, amiodarone, contraceptives, glucocorticoids, lithium, etc.) (4); presence of liver and kidney, respiratory, cardiocerebrovascular, hematological, or autoimmune diseases or tumors (5); presence of pregnancy complications (such as gestational diabetes mellitus, hypertensive disorders of pregnancy, or preeclampsia).

Inclusion criteria for children (1): age 72 ± 1 months (2); guardian consent and willingness to participate in the on-site assessment. Exclusion criteria for children (1): history of perinatal complications (e.g., fetal distress, preterm birth, neonatal asphyxia, kernicterus, hypoxic–ischemic encephalopathy, or neonatal hypoglycemia) or major pediatric conditions that affect neurodevelopment (e.g., encephalitis, meningitis, type 1 diabetes mellitus, or hypothyroidism) (2); positive family history of neurodevelopmental or psychiatric disorders in first-degree relatives.

Mothers were screened and grouped according to trimester-specific pregnancy thyroid function reference ranges previously established by the Peking University International Hospital (25). IH group: FT4 <5^th^ percentile of the first-trimester–specific reference range (13.80 pmol/L), with a normal TSH level (0.12–4.16 uIU/mL) and thyroid peroxidase antibody (TPOAb)–negative status. Euthyroid group: FT4 level 5^th^–97.5^th^ percentile (13.80–23.55 pmol/L), with a normal TSH level and TPOAb-negative status. Euthyroid control women were individually matched to IH cases in a 1:1 ratio based on age (± 2 years) and parity. Finally, 100 mother–child pairs (50 each in the IH and euthyroid groups) were included for the analysis.

### 2.2 Data collection

Maternal demographic and clinical data were obtained at enrollment using questionnaires. The following details were collected: age, parity, gestational age, medical history, history of abnormal pregnancy (miscarriage, premature birth, stillbirth, birth defects, etc.), medications, educational level, household income, and spouse’s educational level. The height and weight of all pregnant women were measured and recorded by trained nurses. The weight (kg) was divided by height squared (m²) to calculate the body mass index (BMI). Samples of maternal venous blood were collected at 6–11 weeks of gestation (mean: 8.16 weeks) for a single measurement of serum TSH, FT4, and TPOAb levels. Enrolled pregnant women were followed up regularly until delivery. Pregnancy outcomes (spontaneous abortion, preterm birth, gestational diabetes mellitus, etc.), delivery timing, mode of delivery, and neonatal information (sex, birth weight, length, and health status) were recorded.

**Figure 1:**
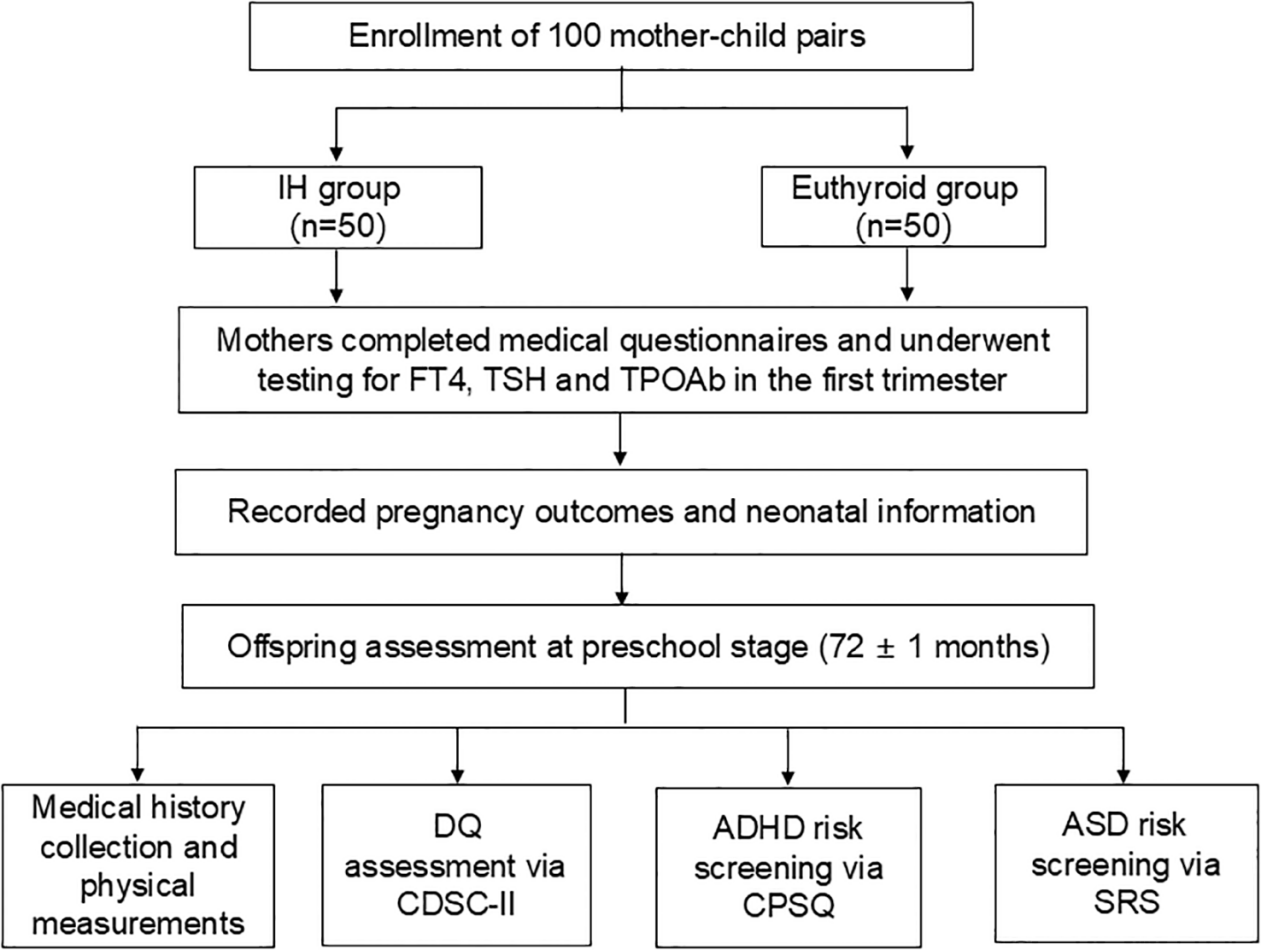
Flow chart of the study. IH, isolated hypothyroxinemia; FT4, free thyroxine; TSH, thyroid stimulating hormone; TPOAb, thyroid peroxidase antibody; DQ, developmental quotient; CDSC-II, Chinese developmental scale for children aged 0–6 Years (2nd Edition); ADHD, attention deficit/hyperactivity disorder; CPSQ, Conners parent symptom questionnaire; ASD, autism spectrum disorder; SRS, social responsiveness scale.

When the children were 72 ± 1 months old, their intellectual development was evaluated using the Chinese Developmental Scale for Children Aged 0–6 Years (2^nd^ Edition) (CDSC-II). A dual-staff protocol was used for the assessment. While participant scheduling and notification were managed by a research coordinator, all formal assessments were administered by a physician who had undergone standardized professional training specific to the CDSC-II. The assessing physician was strictly blinded to maternal thyroid function status during the entire process. The children were screened for ADHD and ASD risk using the Conners Parent Symptom Questionnaire (CPSQ) and the Social Responsiveness Scale (SRS), respectively, by parents (**Figure 1**).

#### 2.2.1 Thyroid function assessment

Serum levels of FT4, TSH, and TPOAb were estimated using a Roche Cobas Elecsys 601 analyzer (Roche, Basel, Switzerland). The intra-assay coefficients of variation (CVs) for TSH, FT4, and TPOAb were 1.1%–3.0%, 1.5%–4.3%, and 2.7%–6.3%, respectively. The corresponding inter-assay CVs were 3.2%–7.2%, 3.3%–8.4%, and 4.2%–9.5%, respectively. The reference range for TPOAb was <34 IU/L.

#### 2.2.2 Neurodevelopmental assessment tools

The CDSC was originally developed in 1980 via a collaborative effort between the Capital Institute of Pediatrics and the Institute of Psychology, Chinese Academy of Sciences. The second edition, which was revised in 2016, was adopted for this research as it is a specialized tool for evaluating early childhood neurodevelopment in China. This scale demonstrates test–retest reliability coefficients in the range of 0.73–0.81 and a validity coefficient of 0.95 (26). The scale comprises 261 items assessing five developmental domains, including gross motor, fine motor, language, adaptive ability, and social behavior. The development quotient (DQ) was used to determine the intellectual development status of the children.

The CPSQ was initially developed by Dr. C. Keith Conners in 1969 and has undergone multiple revisions and improvements. In this study, the version revised by Conners, Goyette, and Ulrich in 1978 was selected because it is the most widely used version in clinical practice. This well-validated scale exhibited robust applicability in screening for ADHD in children (27). The questionnaire comprised 48 items, which were organized into six factors: conduct problems, learning problems, psychosomatic disorders, impulsivity–hyperactivity, anxiety, and hyperactivity index.

SRS, developed by Constantino and Gruber in 2005, was designed to assess the social performance of children and adolescents aged 4–18 years in natural social settings over the past 6 months. It has been extensively employed as a screening tool for ASD, with empirical studies confirming its satisfactory reliability and validity (28). The scale comprises 65 items spanning five domains: social awareness, social cognition, social communication, social motivation, and autistic mannerisms.

### 2.3 Covariates

Potential confounding factors that may affect child neurodevelopment were selected based on previous literature (22, 29). These included gestational age at birth, child sex, maternal age, parity, abnormal pregnancy history, BMI, smoking and alcohol use during early pregnancy, household income, and parental education level.

In this study, maternal age and parity were controlled using a case–control matching design. None of the participating mothers reported a history of smoking or alcohol consumption during early pregnancy. Hence, gestational age at birth, child sex, abnormal pregnancy history, first-trimester BMI, household income, and parental education level were the covariates ultimately adjusted for in the analysis.

### 2.4 Sample size calculation

As intellectual development is a core domain of neurodevelopment, the offspring’s DQ was prespecified as the primary outcome for sample size estimation. Based on prior data (30) (IH group: 87.49 ± 6.34 vs. controls: 95.23 ± 2.75; Cohen’s d = 1.58), a conservative medium independent effect of maternal IH was assumed (Cohen’s f² = 0.15). For a linear regression model adjusting for seven covariates, with α = 0.05 and 90% power, 86 mother–child pairs were required. Accounting for 10% loss to follow-up, the target sample size was set to 100 pairs (50 each in the IH and euthyroid groups, 1:1 ratio).

### 2.5 Statistical analysis

Statistical analyses were conducted using SPSS 20.0 and R 4.2.1 software. The Shapiro-Wilk test was applied to assess the normality of continuous variables. Normally distributed data were presented as mean ± standard deviation. Non-normally distributed data were expressed as median (interquartile range). Categorical variables were presented as frequencies and percentages [n (%)]. Continuous variables were compared using independent samples t-tests (normal distribution) or Mann-Whitney U tests (non-normal distribution). Categorical variables were compared using χ² or Fisher’s exact tests, as appropriate. Linear regression analysis was performed to examine the association between maternal IH during early pregnancy and offspring neurodevelopmental outcomes. In the multiple linear regression model, offspring neurodevelopmental score was set as the outcome variable, with maternal IH status (present vs. absent) as the primary independent variable. All confounding factors were simultaneously incorporated into the model via the forced entry method. To further explore potential sex-specific associations, sex-stratified analyses were conducted and an interaction term between maternal IH and offspring sex was included to assess interaction effects. Additionally, a sensitivity analysis was performed to verify the robustness of the findings. A *P*-value < 0.05 was considered statistically significant. Statistical graphs were generated using GraphPad Prism 10.5 software.

## 3 Results

### 3.1 Comparison of general characteristics between the IH and euthyroid groups

As listed in **Table 1**, offspring in the IH group were more likely to be delivered via cesarean section than those in the euthyroid group (34.00% vs. 8.00%, *P* = 0.001). No statistically significant differences were noted between the two groups in terms of sex distribution, anthropometric measurements at evaluation (height, weight, and head circumference), gestational age, birth weight and length, exclusive breastfeeding for the first 6 months after birth, or primary caregiver before 3 years of age (all *P* > 0.05).

**Table 1:** Comparison of general characteristics between the IH group and the euthyroid group.

Variables	Euthyroid group (n = 50)	IH group (n = 50)	Statistic	*P*
Children’s characteristics				
Sex			χ²=2.56	0.109
Female [n (%)]	22 (44.00)	30 (60.00)		
Male [n (%)]	28 (56.00)	20 (40.00)		
Height at Assessment (cm)	120.00 (118.00, 122.00)	119.00 (116.00, 120.75)	Z=-1.76	0.078
Weight at Assessment (kg)	23.05 (21.40, 24.00)	22.70 (21.30, 24.15)	Z=-0.14	0.885
Head Circumference at Assessment(cm)	51.40 (50.73, 52.08)	51.70 (50.80, 52.10)	Z=-0.89	0.372
Gestational Age at Birth (weeks)	39.15 (38.02, 39.90)	39.25 (38.12, 40.00)	Z=-0.05	0.959
Birth Length (cm)	50.00 (49.00, 51.00)	51.00 (49.00, 51.00)	Z=-1.06	0.286
Birth Weight (g)	3308.40 ± 385.23	3271.80 ± 527.66	t=0.40	0.693
Mode of Delivery			χ²=10.19	0.001
Vaginal Delivery [n (%)]	46 (92.00)	33 (66.00)		
Cesarean Section [n (%)]	4 (8.00)	17 (34.00)		
Exclusive Beastfeeding at 6 Months [n (%)]	26 (52.00)	27 (54.00)	χ²=0.04	0.841
Primary Caregiver before 3 Years of Age			χ²=1.00	0.316
Parental [n (%)]	24 (48.00)	23 (46.00)		
Non-parental [n (%)]	26 (52.00)	27 (54.00)		
Maternal characteristics				
Age at Delivery (years)	31.00 (29.00, 34.00)	31.00(30.00, 34.00)	Z=-0.21	0.830
≥ 35 [n (%)]	12 (24.00)	9 (18.00)	χ²=0.54	0.461
Parity			χ²=0.00	1.000
Nulliparous [n (%)]	15 (30.00)	15 (30.00)		
Multiparous [n (%)]	35 (70.00)	35 (70.00)		
Abnormal Pregnancy History [n (%)]	10 (20.00)	4 (8.00)	χ²=2.99	0.084
Education Level			-	1.000
High School and Below [n (%)]	1 (2.00)	2 (4.00)		
College/University [n (%)]	35 (70.00)	34 (68.00)		
Postgraduate [n (%)]	14 (28.00)	14 (28.00)		
Annual Household Income (CNY)			-	1.000
50,000-100,000 [n (%)]	2 (4.00)	3 (6.00)		
100,000-400,000 [n (%)]	28 (56.00)	27 (54.00)		
≥ 400,000 [n (%)]	20 (40.00)	20 (40.00)		
First-trimester Smoking [n (%)]	0	0	-	-
First-trimester Alcohol Use [n (%)]	0	0	-	-
First-trimester Folate Supplementation [n (%)]	43 (86.00)	42 (84.00)	χ²=0.08	0.779
BMI (kg/m²)	22.34 (20.70, 23.99)	22.15 (20.58, 24.07)	Z=-0.06	0.956
≥ 24 [n (%)]	13 (26.00)	15 (30.00)	χ²=0.20	0.656
FT4 (pmol/L)	16.60 (15.89, 17.62)	12.80 (11.72, 13.30)	Z=-8.60	< 0.001
TSH (uIU/ml)	1.56 (0.92, 2.48)	2.11 (1.57, 2.80)	Z=-2.53	0.011
Paternal characteristics				
Age at Delivery (years)	32.50 (30.00, 35.00)	32.00 (30.00, 35.00)	Z=-0.32	0.750
Education Level			χ²=0.17	0.680
College/University [n (%)]	30 (60.00)	32 (64.00)		
Postgraduate [n (%)]	20 (40.00)	18 (36.00)		
First-trimester Smoking [n (%)]	11 (22.00)	14 (28.00)	χ²=0.48	0.488
First-trimester Alcohol Use [n (%)]	14 (28.00)	19 (38.00)	χ²=1.13	0.288

IH, isolated hypothyroxinemia; CNY, China yuan; BMI, body mass index; FT4, free thyroxine; TSH, thyroid stimulating hormone.

Mothers in the IH group had significantly lower FT4 levels (*P* < 0.001) and higher TSH levels (*P* = 0.001) than those in the euthyroid group. No statistically significant differences were observed between the two groups of mothers regarding age at delivery, parity, adverse pregnancy history, educational level, annual family income, smoking/alcohol use, folate supplementation, or BMI in early pregnancy (all *P* > 0.05).

Compared with fathers in the euthyroid group, those in the IH group did not exhibit significant differences in age at offspring birth, educational level, or smoking and alcohol consumption during early pregnancy (all *P* > 0.05).

### 3.2 Comparison of assessment results of the offspring

**Figure 2:**
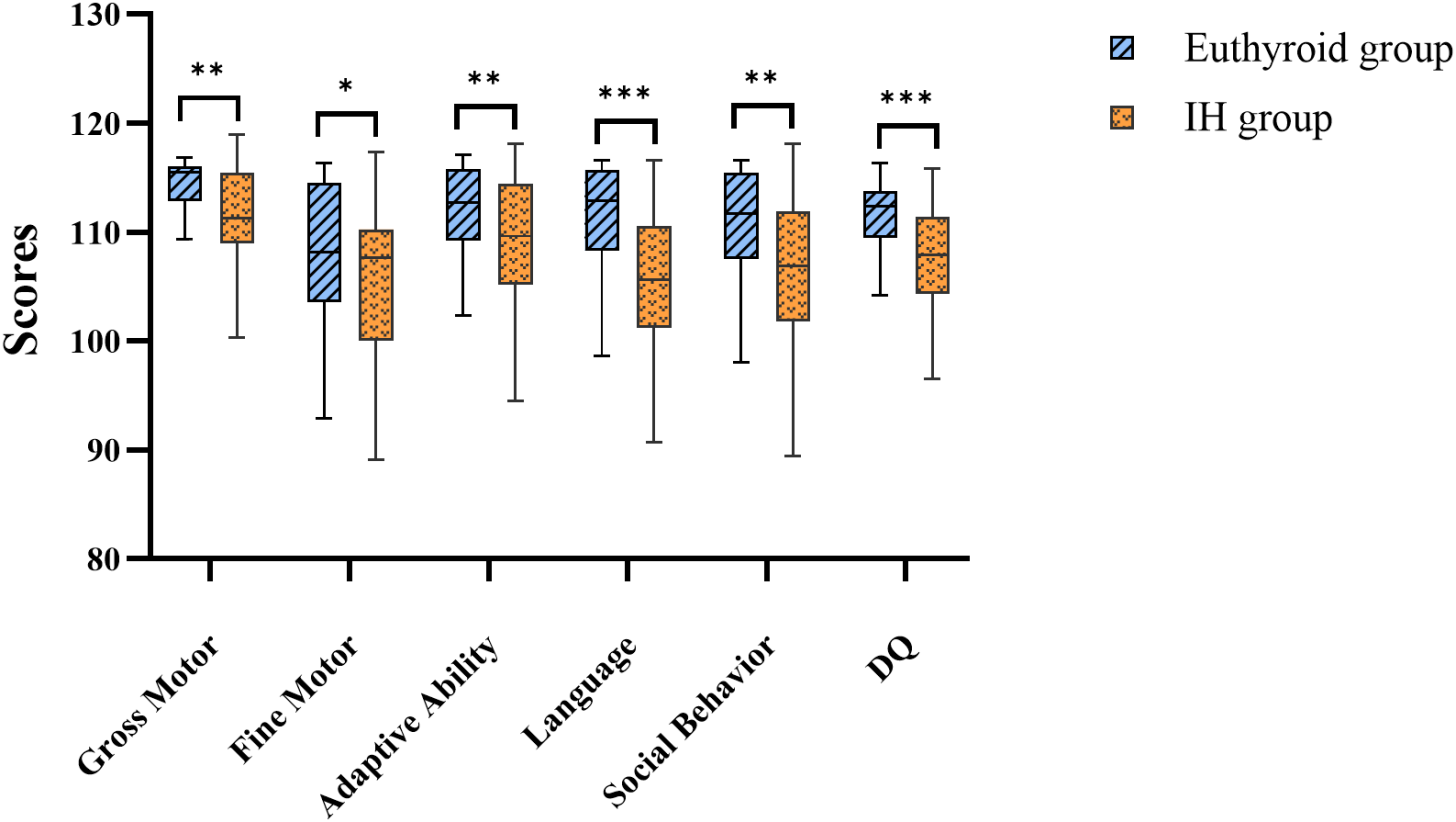
Comparison of offspring’s CDSC-II scores. IH, isolated hypothyroxinemia; DQ, development quotient; CDSC-II, Chinese developmental scale for children aged 0–6 Years (2nd Edition). Ns, not significant; *P < 0.05; **P < 0.01; ***P < 0.001.

The findings from CDSC-II revealed that offspring in the IH group exhibited significantly lower DQ than those in the euthyroid group (*P* < 0.05). In addition, scores in all five domains (gross motor, fine motor, adaptive ability, language, and social behavior) were lower in the IH group compared with those in the euthyroid group (all *P* < 0.05). (**Figure 2**; **Supplementary Material Table 1S**).

**Figure 3:**
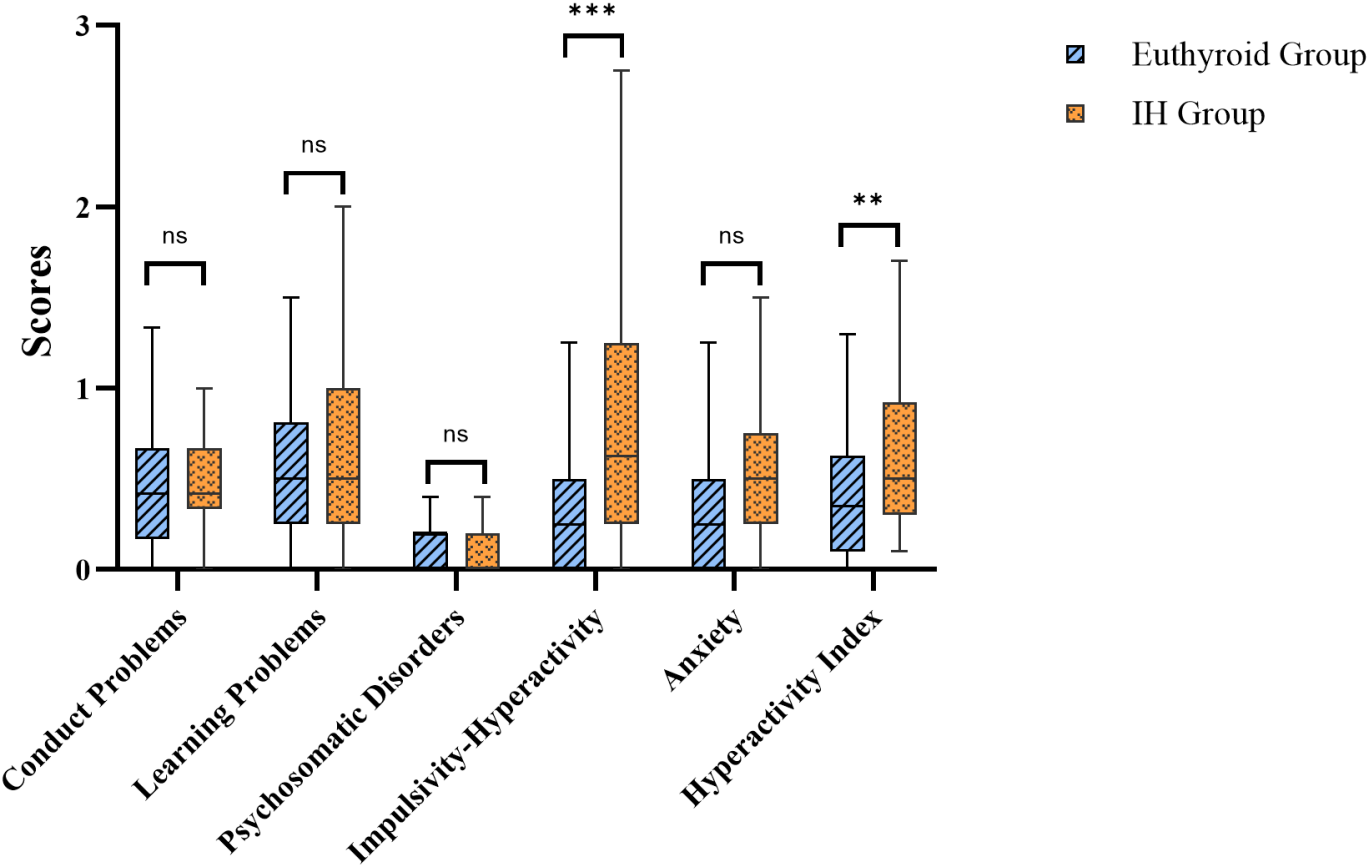
Comparison of offspring’s CPSQ scores. IH, isolated hypothyroxinemia; CPSQ, Conners parent symptom questionnaire. Ns, not significant; **P < 0.01; ***P < 0.001.

The observations from CPSQ signified that offspring in the IH group displayed higher scores than those in the euthyroid group on both the impulsivity–hyperactivity subscale (*P* < 0.001) and the hyperactivity index (*P* = 0.003). Statistically significant differences were not evident between the two groups in the scores of the offspring for conduct problems, learning problems, psychosomatic disorders, or anxiety dimensions (all *P* > 0.05) (**Figure 3**; **Supplementary Material Table 2S**).

**Figure 4:**
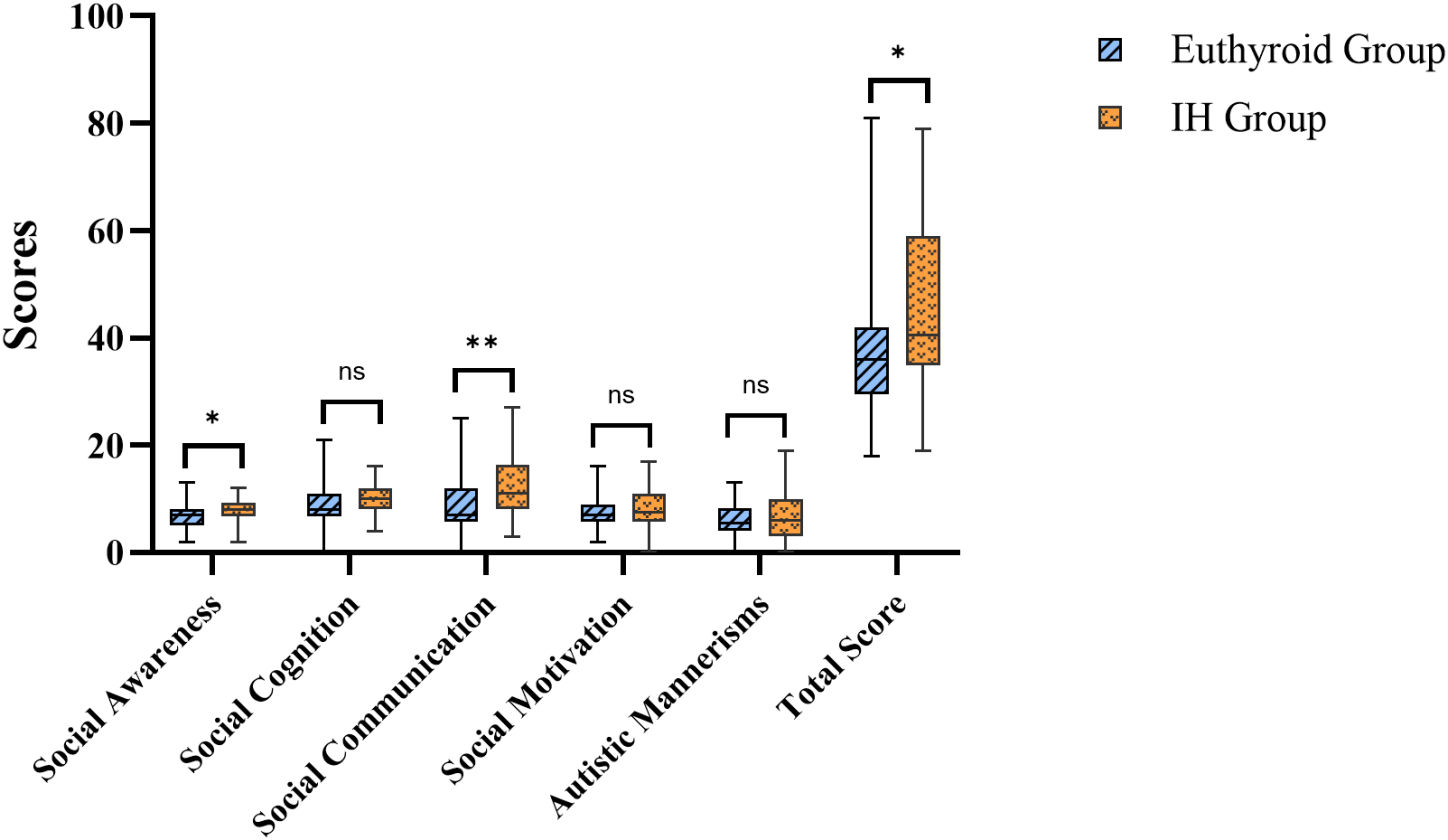
Comparison of offspring’s SRS scores. IH, isolated hypothyroxinemia; SRS, social responsiveness scale. Ns, not significant; *P < 0.05; **P < 0.01.

According to the SRS results, compared with offspring in the euthyroid group, those in the IH group demonstrated higher total scores and subscale scores in social awareness and social communication. No statistically significant differences were perceived in social cognition, social motivation, or autism mannerisms between offspring in the two groups (all *P* > 0.05) (**Figure 4**; **Supplementary Material Table 3S**).

### 3.3 Association between first-trimester maternal IH and offspring neurodevelopmental outcomes

**Figure 5:**
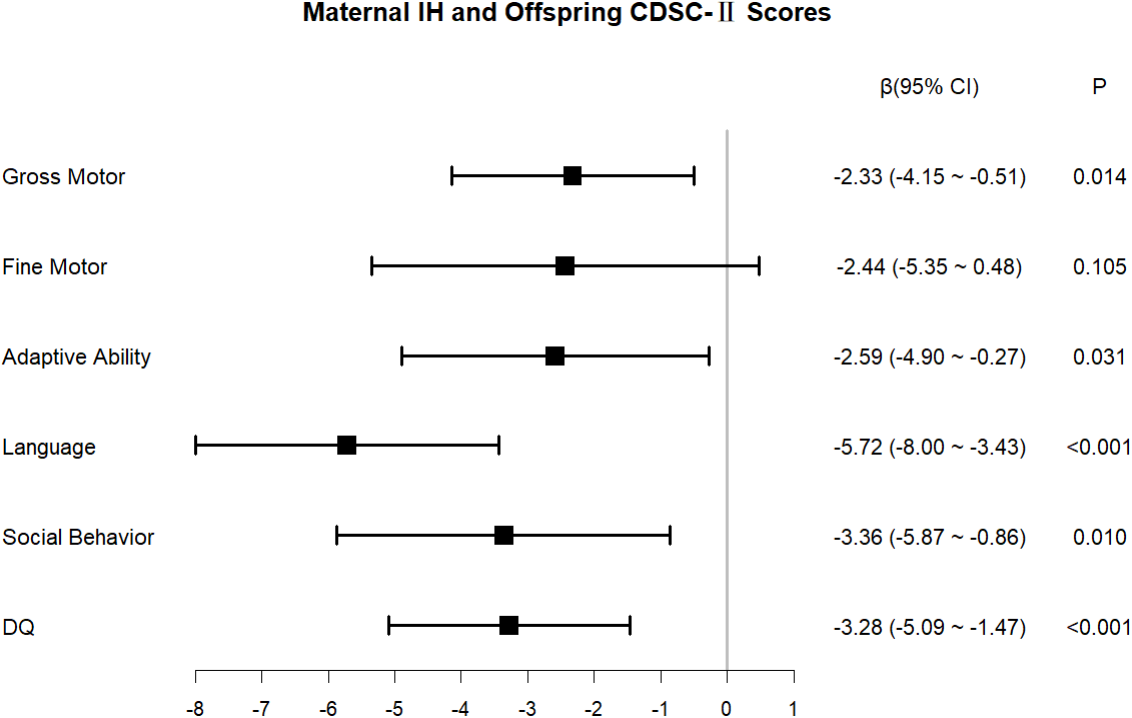
Association between maternal IH in the first trimester and offspring’s CDSC-II scores. IH, isolated hypothyroxinemia; CDSC-II, Chinese developmental scale for children aged 0–6 Years (2nd Edition); DQ, development quotient.

**Figure 6:**
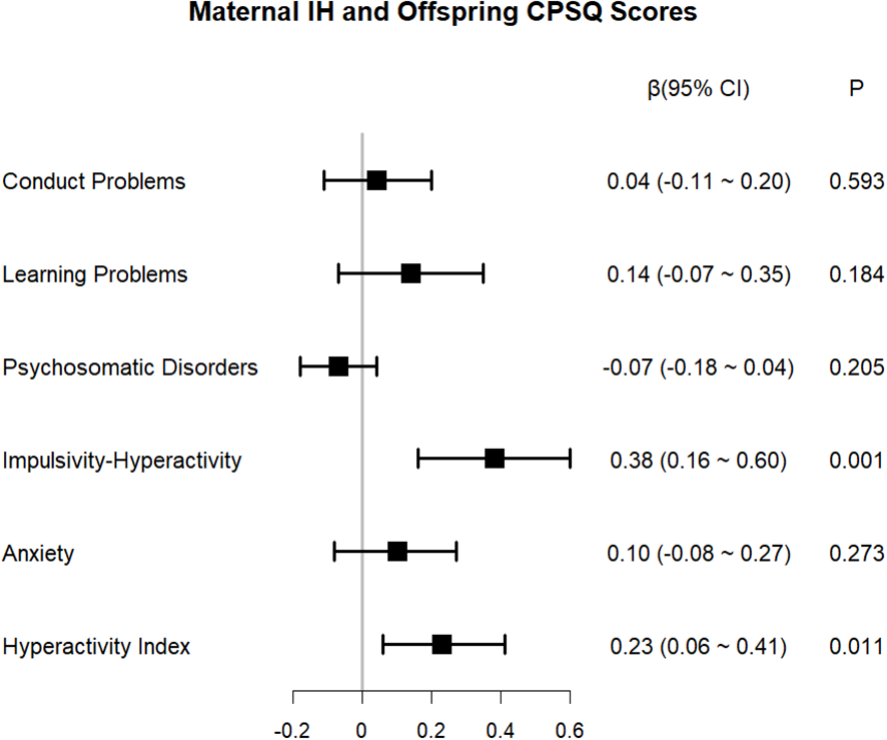
Association between maternal IH in the first trimester and offspring’s CPSQ scores. IH, isolated hypothyroxinemia; CPSQ, Conners parent symptom questionnaire.

**Figure 7:**
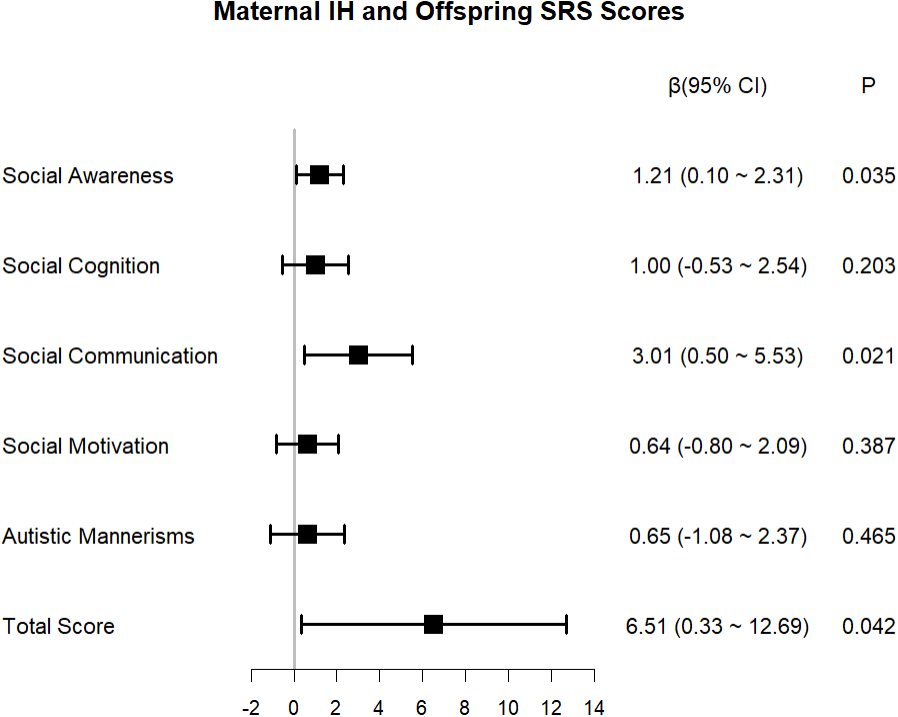
Association between maternal IH in the first trimester and offspring’s SRS scores. IH, isolated hypothyroxinemia; SRS, social responsiveness scale.

The findings from simple and multiple linear regression are furnished in **Supplementary Material Table 4S**. The predefined covariates listed in the Methods section (gestational age at birth, child sex, maternal history of abnormal pregnancy, first-trimester BMI, household income, and parental education) were adjusted for in the multiple regression model. Furthermore, the mode of delivery, which showed significant intergroup differences in univariable analysis, was included in the model. The results indicated a negative association between maternal IH in the first trimester and the DQ of the offspring (adjusted β = −3.28, 95% CI: −5.09 to −1.47, *P* < 0.001). Domain-specific analyses revealed that maternal IH in the first trimester exerted the greatest negative effect on children’s language development (adjusted β = −5.72, 95% CI: −8.00 to −3.43, *P* < 0.001), followed by social behavior (adjusted β = −3.36, 95% CI: −5.87 to −0.86, *P* = 0.010), adaptive ability (adjusted β = −2.59, 95% CI: −4.90 to −0.27, *P* = 0.031), and gross motor skills (adjusted β = −2.33, 95% CI: −4.15 to −0.51, *P* = 0.014). The association between maternal IH in the first trimester and the offspring’s fine motor skills failed to attain statistical significance (adjusted β = −2.44, 95% CI: −5.35 to 0.48, *P* = 0.105) (**Figure 5**). As depicted in **Figure 6**, maternal IH during early pregnancy was positively linked to increased impulsive–hyperactive (adjusted β = 0.38, 95% CI: 0.16–0.60, *P* = 0.001) and hyperactivity index (adjusted β = 0.23, 95% CI: 0.06–0.41, *P* = 0.011) scores in the offspring. However, conduct problems, learning problems, psychosomatic disorders, or anxiety did not exhibit significant associations (all *P* > 0.05). As illustrated in **Figure 7**, maternal IH in the first trimester was positively associated with higher scores in the social awareness of the offspring (adjusted β = 1.21, 95% CI: 0.10–2.31, *P* = 0.035), social communication (adjusted β = 3.01, 95% CI: 0.50–5.53, *P* = 0.021), and the total score (adjusted β = 6.51, 95% CI: 0.33–12.69, *P* = 0.042). Maternal first-trimester IH did not exhibit a statistically significant association with the offspring’s social cognition, social motivation, or autistic mannerisms (all *P* > 0.05).

### 3.4 Sex-specific associations between first-trimester maternal IH and offspring neurodevelopmental outcomes

To determine sex-specific patterns in the link between first-trimester maternal IH and offspring neurodevelopmental outcomes, sex-stratified analyses were conducted, and the IH-by-sex interaction was tested (**Supplementary Material Table S5**). In male offspring (n = 48), maternal IH was correlated with lower scores in gross motor, adaptive ability, language, social behavior, and DQ on the CDSC-II scale and higher scores in impulsivity–hyperactivity and the hyperactivity index on the CPSQ scale (all *P* < 0.05). In contrast, in the female offspring (n = 52), significant associations were noted only for lower CDSC-II language scores and higher CPSQ impulsivity–hyperactivity scores (both *P* < 0.05). Despite these differences in stratified associations, the interaction between IH and offspring sex was not statistically significant for any neurodevelopmental outcome (all *P* > 0.05).

### 3.5 Sensitivity analysis

As presented in **Supplementary Material Table 6S**, sensitivity analysis performed by excluding the mode of delivery from the regression models established that the core findings remained consistent in both the total population and the sex-stratified subgroups (boys and girls).

## 4 Discussion

Previous research on the link between maternal IH during pregnancy and offspring neuropsychiatric development has yielded inconsistent results. Being a prospective cohort study focused on the Chinese population, this investigation is the first to perform comprehensive assessments within the same cohort at a key developmental stage—the preschool years (72 ± 1 months)—to identify neurodevelopmental risks. Besides evaluating cognitive domains using CDSC-II, CPSQ, and SRS were incorporated to screen for ADHD and ASD. The assessment tools indicated strong reliability and validity in the Chinese population, strengthening confidence in the obtained results. The IH diagnosis was strictly based on pregnancy-specific thyroid function reference ranges derived from local pregnant women, which considerably augmented the diagnostic accuracy. Moreover, various confounding factors, including the mode of delivery (a higher rate of cesarean section was observed in the IH group), gestational age, child sex, maternal pregnancy history, first-trimester BMI, household income, and parental education, were adjusted for. Sensitivity analyses that excluded the mode of delivery were performed to confirm the robustness of the findings. This study identified that maternal IH during the first trimester was associated with lower DQ in children, substantially affecting language, social behavior, and adaptive skills. In addition, maternal IH was linked to increased impulsivity–hyperactivity scores, a higher hyperactivity index, and elevated SRS total and subscale scores, particularly in social awareness and communication. While sex-specific analysis hinted that certain effects might be more pronounced in boys, a statistically significant interaction was not evident between IH and the child’s sex.

Maternal THs provide major support for fetal brain development in early pregnancy. Even mild maternal TH deficiency during this crucial period can negatively affect the child’s neurodevelopment. IH is a common thyroid disorder during pregnancy. In a groundbreaking prospective study involving 220 mother–child pairs (which included 33 mothers with IH), Pop et al. found that first-trimester maternal IH (defined as FT4 <10^th^ percentile at 12 weeks) was correlated with lower psychomotor and mental scores on the Bayley Scales at 10 months and 2 years of age (17, 31). This study observed that maternal first-trimester IH was independently associated with lower offspring DQ (adjusted β = −3.28; 95% CI: −5.09 to −1.47; *P* < 0.001). This finding largely agrees with certain previous investigations (18, 19, 32–34). In contrast, studies evaluating maternal THs during mid-gestation found no significant links between IH and the cognitive outcomes of the offspring (35–37). These differing findings underscore the critical role of gestational timing in determining the neurodevelopmental impact of maternal IH. Early gestation is a period of rapid fetal neuronal proliferation and migration, during which the fetus depends entirely on maternal TH supply. Conversely, mid-gestation marks the onset of fetal thyroid function, potentially mitigating the effects of maternal TH deficiency. Maternal IH potentially contributes to offspring cognitive impairment via multiple mechanisms: persistent DNA hypermethylation of the hippocampal brain-derived neurotrophic factor gene (38), reduced phosphorylation of glutamate receptor 1 in the hippocampal CA1 region (39), and disrupted tangential migration of medial ganglionic eminence–derived neurons in the cerebral cortex.

A previous study documented that maternal IH during pregnancy might be linked to delays in cognitive and motor development in children as well as elevated risks of psychiatric conditions. More recent studies have focused on the connection between maternal IH and the risk of ADHD in the offspring, but the findings remain inconclusive. An early study by Vermiglio et al. in iodine-deficient regions reported a significantly higher ADHD risk at ages 8–10 years among children of mothers with IH in the first half of pregnancy (40). Later research in iodine-sufficient populations also identified associations between early-pregnancy IH and increased ADHD symptoms (20, 21). In contrast, two large prospective studies (41, 42) and a 2019 individual participant data (IPD) meta-analysis pooling 7,669 mother–child pairs from three European cohorts failed to find a link between maternal FT4 levels during early pregnancy and offspring ADHD (43). Nonetheless, two subsequent meta-analyses observed that maternal IH significantly increased ADHD risk in the offspring (44, 45). Sex-stratified analyses have also yielded conflicting results. Andersen et al. (42) reported a significant association exclusively in the female offspring (adjusted HR: 2.3, 95% CI: 1.2–4.3, *P* < 0.05) (46). On the contrary, Gong et al. observed that first-trimester maternal IH was specifically correlated with a higher hyperactivity index in the male offspring (4.7 ± 2.9 vs. 2.7 ± 1.6, *P* < 0.05) but not in the female offspring (3.5 ± 2.2 vs. 2.8 ± 1.8, *P* > 0.05) (47). This study found a positive link between maternal IH in the first trimester and higher impulsivity–hyperactivity scores and hyperactivity index in children. These results do not fully align with earlier research. The differences could be attributed to various factors, including the timing of FT4 measurement, study populations (such as ethnicity), iodine nutritional status, assessment methods, and the covariates included in the analysis. The precise mechanism by which maternal IH affects ADHD risk in the offspring remains unknown. An animal study (47) revealed that male offspring of rats exposed to gestational IH exhibited ADHD-like behaviors. The possible underlying mechanism involves TH deficiency, leading to the dysregulated expression of critical neurochemical markers in the offspring. These include dopamine D1 receptors in the prefrontal cortex and cerebellum, synaptosomal-associated protein 25 kDa in the cerebral cortex and hippocampus, and monoamine oxidase in the striatum.

Emerging evidence indicates that maternal IH during pregnancy could be linked to a higher risk of ASD in children, although studies are still limited. This hypothesis was initially proposed by Román et al. in 2007, citing neuroanatomical similarities between ASD and brain abnormalities seen in offspring exposed to maternal IH (48). Subsequently, a prospective study of the Generation R cohort by Román et al. in 2013 found that offspring of mothers with severe IH (FT4 < 5^th^ percentile, n = 136) in early-mid gestation (6–18 weeks) exhibited a significantly increased ASD risk at 6 years of age (adjusted OR: 3.89, 95% CI: 1.83–8.20, *P* < 0.001) but those of mothers with mild IH (FT4 < 10^th^ percentile) did not (22). Andersen et al. (46) observed that maternal IH (FT4 < 2.5^th^ percentile) at 5–19 weeks of gestation exacerbated the risk of ASD in the female offspring but not in the male offspring. An IPD meta-analysis showed that maternal FT4 < 5^th^ percentile in early pregnancy was associated with an increased risk of ASD in children (OR: 1.5, 95% CI: 1.0–2.3, *P* = 0.08). However, FT4 < 2.5^th^ percentile demonstrated no such association risk (23). This study found that maternal IH during the first trimester was linked to higher social awareness and social communication scores, as well as overall SRS scores in the offspring. No differences based on sex were noted, contrasting with earlier research. This variation could be explained by differences in the time of assessment of gestational IH, the diagnostic criteria for IH, and the tools used to measure ASD-related traits. A 2024 animal study provided the first experimental proof that maternal gestational IH causes ASD-like features in the offspring. The study highlighted that dysregulated inflammatory responses and glutamatergic signaling pathways were the key mechanisms. Furthermore, it revealed that T4 supplementation during pregnancy could effectively reduce these ASD-like traits in the offspring (49).

Data from this study showed that cesarean section rates were significantly higher in the IH group than in the euthyroid group (34.00% vs. 8%, *P* = 0.001). To control for the potential confounding influence of the delivery method, it was included as a covariate in the main multivariable analysis. Furthermore, a sensitivity analysis was performed, which excluded this variable altogether. In both cases, the association between IH and the outcomes studied remained consistent in both direction and significance, asserting the robustness of the primary findings from the study.

This prospective cohort study enhances current evidence by including comprehensive developmental assessments, diagnostic criteria tailored to the population, culturally adapted evaluation tools, and rigorous statistical methods. This research provides robust and representative data from a Chinese population, enriching our understanding of the long-term neurodevelopmental effects of IH in early pregnancy on children. Nonetheless, certain limitations should be acknowledged. First, the single-center design and the modest sample size may restrict the applicability of the findings. Second, on-site standardized assessments might have inadvertently excluded children with severe neurodevelopmental symptoms, potentially underestimating the prevalence of impairment. Third, the outcomes relied on screening tools instead of gold-standard clinical diagnoses, reflecting risk factors rather than confirmed disorders. Fourth, indications for cesarean delivery were not recorded. Although the mode of delivery was adjusted for and the robustness of the results was confirmed via sensitivity analyses, residual confounding is a possibility. A more critical limitation is the lack of direct measurement of maternal iodine status, which is essential for both maternal TH synthesis and offspring neurodevelopment. This limitation is partially mitigated by the fact that all participants are long-term residents of Beijing, an iodine-sufficient region. Future studies involving larger, multicenter cohorts with direct iodine measurements, cesarean delivery indications, and combined screening and diagnostic assessments are needed to validate the present findings.

## 5 Conclusion

This study shows that maternal IH in the first trimester is associated with lower scores in the offspring’s intellectual development and higher scores on screening scales for ADHD- and ASD-related symptoms. The results emphasize the significance of maintaining adequate maternal TH levels early in pregnancy for proper fetal brain development. In addition, this study underscores the need for routine thyroid function testing during the first trimester, coupled with timely and effective treatment of thyroid issues for the long-term protection of neurodevelopmental health in children. Larger, multicenter prospective studies are necessary to better understand the underlying mechanisms and to develop optimal therapeutic approaches, ensuring better health outcomes for both mothers and their children.

## Data Availability

The raw data supporting the conclusions of this article will be made available by the authors, without undue reservation.
