# Supplementary material for: Effect of maternal isolated hypothyroxinemia in the first trimester on offspring neurodevelopment: a prospective cohort study
Source: Front Endocrinol (Lausanne). 2026 Feb 18;17:1734708. doi: 10.3389/fendo.2026.1734708 (PMC12957080; doi:10.3389/fendo.2026.1734708)
Supplement: Supplementary file 1 [file Table1.docx]

| **Table 1S Comparison of offspring’s** **CDSC-II scores** | | | | |
| --- | --- | --- | --- | --- |
|  | **Euthyroid group**  **(n = 50)** | **IH group**  **(n = 50)** | **Statistic** | ***P*** |
| Gross Motor | 115.50 (112.98, 116.00) | 111.30 (108.90, 115.27) | Z=-2.70 | 0.007 |
| Fine Motor | 108.15 (103.55, 114.10) | 107.65 (100.72, 110.12) | Z=-2.52 | 0.012 |
| Adaptive Ability | 113.55 (109.70, 115.77) | 109.60 (105.40, 114.03) | Z=-2.59 | 0.010 |
| Language | 112.80 (109.10, 115.65) | 105.55 (101.35, 110.20) | Z=-5.02 | <0.001 |
| Social Behavior | 111.70 (107.55, 115.45) | 106.85 (101.85, 111.58) | Z=-3.01 | 0.003 |
| DQ | 112.40 (109.62, 113.70) | 107.85 (104.40, 111.18) | Z=-4.00 | <0.001 |
| **Abbreviations:** IH, isolated hypothyroxinemia; CDSC-II, Chinese developmental scale for children aged 0-6 Years (2^nd^ Edition); DQ, development quotient | | | | |

| **Table 2S Comparison of** **offspring’s CPSQ scores** | | | | |
| --- | --- | --- | --- | --- |
|  | **Euthyroid group**  **(n = 50)** | **IH group**  **(n = 50)** | **Statistic** | **P** |
|  |  |  |  |  |
| Conduct Problems | 0.42 (0.17, 0.67) | 0.42 (0.33, 0.67) | Z=-1.26 | 0.209 |
| Learning Problems | 0.50 (0.25, 0.75) | 0.50 (0.25, 1.00) | Z=-1.16 | 0.247 |
| Psychosomatic Disorders | 0.20 (0.00, 0.20) | 0.00 (0.00, 0.20) | Z=-0.80 | 0.422 |
| Impulsivity-Hyperactivity | 0.25 (0.00, 0.50) | 0.62 (0.25, 1.19) | Z=-3.80 | <0.001 |
| Anxiety | 0.25 (0.00, 0.50) | 0.50 (0.25, 0.75) | Z=-1.76 | 0.078 |
| Hyperactivity Index | 0.35 (0.10, 0.60) | 0.50 (0.30, 0.90) | Z=-2.79 | 0.005 |
| **Abbreviations:** IH, isolated hypothyroxinemia; CPSQ, Conners parent symptom questionnaire | | | | |

| **Table 3S Comparison of** **offspring’s SRS scores** | | | | |
| --- | --- | --- | --- | --- |
|  | **Euthyroid group**  **(n = 50)** | **IH group**  **(n = 50)** | **Statistic** | ***P*** |
|  |  |  |  |  |
| Social Awareness | 7.00 (5.00, 8.00) | 8.00 (7.00, 9.00) | Z=-2.44 | 0.015 |
| Social Cognition | 8.00 (7.00, 11.00) | 10.00 (8.00, 11.75) | Z=-1.79 | 0.073 |
| Social Communication | 7.00 (6.00, 12.00) | 11.00 (8.00, 16.00) | Z=-2.71 | 0.007 |
| Social Motivation | 7.00 (6.00, 9.00) | 7.50 (6.00, 10.75) | Z=-1.37 | 0.172 |
| Autistic Mannerisms | 5.50 (4.00, 8.00) | 6.00 (3.00, 9.75) | Z=-0.78 | 0.434 |
| Total Score | 36.00 (30.25, 41.50) | 40.50 (35.00, 57.75) | Z=-2.44 | 0.015 |
| **Abbreviations:** IH, isolated hypothyroxinemia; SRS, social responsiveness scale | | | | |

| **Table 4S Linear regression analysis of the association between first-trimester maternal IH and offspring neurodevelopmental outcomes** | | | | |
| --- | --- | --- | --- | --- |
|  | **Model 1** | | **Model 2** | |
|  | **Crude β(95% CI)** | ***P*** | **Adjustedβ(95% CI)** | ***P*** |
| **CDSC-II Scores** | | | | |
| Gross Motor | -2.44 (-4.09 ~ -0.80) | 0.004 | -2.33 (-4.15 ~ -0.51) | 0.014 |
| Fine Motor | -3.51 (-6.13 ~ -0.88) | 0.010 | -2.44 (-5.35 ~ 0.48) | 0.105 |
| Adaptive Ability | -3.30 (-5.37 ~ -1.22) | 0.002 | -2.59 (-4.90 ~ -0.27) | 0.031 |
| Language | -6.24 (-8.31 ~ -4.16) | <0.001 | -5.72 (-8.00 ~ -3.43) | <0.001 |
| Social Behavior | -4.07 (-6.41 ~ -1.74) | <0.001 | -3.36 (-5.87 ~ -0.86) | 0.010 |
| DQ | -3.90 (-5.55 ~ -2.25) | <0.001 | -3.28 (-5.09 ~ -1.47) | <0.001 |
| **CPSQ Scores** | | | | |
| Conduct Problems | 0.07 (-0.06 ~ 0.21) | 0.301 | 0.04 (-0.11 ~ 0.20) | 0.593 |
| Learning Problems | 0.15 (-0.03 ~ 0.33) | 0.114 | 0.14 (-0.07 ~ 0.35) | 0.184 |
| Psychosomatic Disorders | -0.04 (-0.14 ~ 0.06) | 0.438 | -0.07 (-0.18 ~ 0.04) | 0.205 |
| Impulsivity-Hyperactivity | 0.40 (0.19 ~ 0.60) | <0.001 | 0.38 (0.16 ~ 0.60) | 0.001 |
| Anxiety | 0.12 (-0.04 ~ 0.28) | 0.133 | 0.10 (-0.08 ~ 0.27) | 0.273 |
| Hyperactivity Index | 0.24 (0.08 ~ 0.40) | 0.003 | 0.23 (0.06 ~ 0.41) | 0.011 |
| **SRS Score** |  |  |  |  |
| Social Awareness | 1.18 (0.10 ~ 2.06) | 0.034 | 1.21 (0.10 ~ 2.31) | 0.035 |
| Social Cognition | 0.98 (-0.38 ~ 2.34) | 0.160 | 1.00 (-0.53 ~ 2.54) | 0.203 |
| Social Communication | 3.04(0.79 ~ 5.29) | 0.010 | 3.01 (0.50 ~ 5.53) | 0.021 |
| Social Motivation | 1.02 (-0.32 ~ 2.36) | 0.138 | 0.64 (-0.80 ~ 2.09) | 0.387 |
| Autistic Mannerisms | 1.00 (-0.53 ~ 2.53) | 0.203 | 0.65 (-1.08 ~ 2.37) | 0.465 |
| Total Score | 7.12 (1.59 ~ 12.65) | 0.013 | 6.51 (0.33 ~ 12.69) | 0.042 |
| **Model 1:** none (univariable); **Model 2:** adjusted for gestational age at birth, child sex, maternal abnormal pregnancy history, first-trimester BMI, household income, parental education and mode of delivery  **Abbreviations:** IH, isolated hypothyroxinemia; CDSC-II, Chinese developmental scale for children aged 0-6 Years (2^nd^ Edition); DQ, development quotient; CPSQ, Conners parent symptom questionnaire; SRS, social responsiveness scale; CI, Confidence Interval | | | | |

| **Table 5S Sex-specific associations between first-trimester maternal IH and offspring neurodevelopmental outcomes** | | | | | |
| --- | --- | --- | --- | --- | --- |
|  | **Boyes (n=48)** | | **Girls (n=52)** | | **Interaction** |
|  | **β (95% CI)** | ***P*** | **β (95% CI)** | ***P*** | ***P*** |
| **CDSC-II Scores** |  |  |  |  |  |
| Gross Motor | -3.18 (-5.79 ~ -0.57) | 0.022 | -1.55 (-4.55 ~ 1.45) | 0.316 | 0.220 |
| Fine Motor | -2.83 (-7.27 ~ 1.60) | 0.219 | -2.08 (-6.00 ~ 1.85) | 0.306 | 0.954 |
| Adaptive Ability | -3.84 (-6.77 ~ -0.91) | 0.014 | -1.70 (-5.60 ~ 2.19) | 0.396 | 0.344 |
| Language | -7.35 (-10.80 ~ -3.90) | <0.001 | -4.67 (-8.29 ~ -1.05) | 0.015 | 0.289 |
| Social Behavior | -4.57 (-7.52 ~ -1.62) | 0.004 | -2.14 (-6.62 ~ 2.35) | 0.356 | 0.700 |
| DQ | -4.33 (-6.64 ~ -2.02) | <0.001 | -2.45 (-5.50 ~ 0.59) | 0.122 | 0.416 |
| **CPSQ Scores** |  |  |  |  |  |
| Conduct Problems | 0.07 (-0.17 ~ 0.31) | 0.565 | 0.06 (-0.18 ~ 0.31) | 0.608 | 0.884 |
| Learning Problems | 0.17 (-0.19 ~ 0.53) | 0.361 | 0.09 (-0.20 ~ 0.38) | 0.534 | 0.892 |
| Psychosomatic Disorders | 0.01 (-0.15 ~ 0.17) | 0.893 | -0.13 (-0.31 ~ 0.06) | 0.188 | 0.718 |
| Impulsivity-Hyperactivity | 0.60 (0.26 ~ 0.94) | 0.002 | 0.32 (0.03 ~ 0.61) | 0.035 | 0.923 |
| Anxiety | 0.22 (-0.06 ~ 0.50) | 0.129 | 0.11 (-0.16 ~ 0.38) | 0.417 | 0.774 |
| Hyperactivity Index | 0.32 (0.03 ~ 0.62) | 0.040 | 0.17 (-0.07 ~ 0.41) | 0.185 | 0.784 |
| **SRS Score** |  |  |  |  |  |
| Social Awareness | 0.39 (-1.35 ~ 2.12) | 0.666 | 1.73 (-0.13 ~ 3.59) | 0.071 | 0.359 |
| Social Cognition | 1.72 (-0.96 ~ 4.40) | 0.216 | 0.55 (-1.50 ~ 2.60) | 0.602 | 0.954 |
| Social Communication | 3.06 (-0.76 ~ 6.88) | 0.125 | 2.75 (-1.01 ~ 6.51) | 0.159 | 0.681 |
| Social Motivation | 0.63 (-1.79 ~ 3.04) | 0.614 | 1.06 (-1.01 ~ 3.13) | 0.321 | 0.763 |
| Autistic Mannerisms | -0.39 (-2.98 ~ 2.20) | 0.769 | 0.72 (-1.87 ~ 3.31) | 0.588 | 0.269 |
| Total Score | 5.40 (-4.27 ~ 15.06) | 0.281 | 6.82 (-2.10 ~ 15.74) | 0.142 | 0.488 |
| **Model:** adjusted for gestational age at birth, maternal abnormal pregnancy history, first-trimester BMI, household income, parental education and mode of delivery  **Abbreviations:** IH, isolated hypothyroxinemia; CDSC-II, Chinese developmental scale for children aged 0-6 Years (2^nd^ Edition); DQ, development quotient; CPSQ, Conners parent symptom questionnaire; SRS, social responsiveness scale; CI, Confidence Interval | | | | | |

| **Table 6S Sensitivity analysis of association between first-trimester maternal IH and offspring neurodevelopmental outcomes** | | | | | | |
| --- | --- | --- | --- | --- | --- | --- |
|  | **Total (n=100**) | | **Boyes (n=48)** | | **Girls (n=52)** | |
|  | **β (95% CI)** | ***P*** | **β (95% CI)** | ***P*** | **β (95% CI)** | ***P*** |
| **CDSC-II Scores** | | | | | | |
| Gross Motor | -2.57 (-4.29 ~ -0.85) | 0.004 | -3.91 (-6.22 ~ -1.60) | 0.002 | -1.53 (-4.46 ~ 1.39) | 0.309 |
| Fine Motor | -2.72 (-6.03 ~ 0.59) | 0.076 | -3.68 (-7.58 ~ 0.21) | 0.071 | -2.63 (-6.57 ~ 1.31) | 0.198 |
| Adaptive Ability | -3.20 (-5.42 ~ -0.98) | 0.006 | -4.29 (-6.85 ~ -1.73) | 0.002 | -2.00 (-5.83 ~ 1.83) | 0.312 |
| Language | -5.98 (-8.15 ~ -3.82) | <0.001 | -7.23 (-10.23 ~ -4.23) | <0.001 | -4.88 (-8.43 ~ -1.34) | 0.010 |
| Social Behavior | -3.99 (-6.38 ~ -1.59) | 0.002 | -4.63 (-7.19 ~ -2.07) | 0.001 | -2.72 (-7.20 ~ 1.77) | 0.241 |
| DQ | -3.82 (-5.56 ~ -2.08) | <0.001 | -4.71 (-6.73 ~ -2.69) | <0.001 | -2.78 (-5.80 ~ 0.24) | 0.079 |
| **CPSQ Scores** | | | | | | |
| Conduct Problems | 0.06 (-0.09 ~ 0.21) | 0.422 | 0.10 (-0.11 ~ 0.30) | 0.362 | 0.08 (-0.16 ~ 0.32) | 0.504 |
| Learning Problems | 0.16 (-0.04 ~ 0.35) | 0.120 | 0.20 (-0.11 ~ 0.51) | 0.214 | 0.09 (-0.20 ~ 0.37) | 0.556 |
| Psychosomatic Disorders | -0.04 (-0.15 ~ 0.07) | 0.482 | 0.02 (-0.12 ~ 0.16) | 0.779 | -0.10 (-0.29 ~ 0.09) | 0.324 |
| Impulsivity-Hyperactivity | 0.37 (0.16 ~ 0.58) | <0.001 | 0.52 (0.22 ~ 0.83) | 0.001 | 0.33 (0.05 ~ 0.60) | 0.028 |
| Anxiety | 0.11 (-0.05 ~ 0.27) | 0.194 | 0.14 (-0.11 ~ 0.39) | 0.271 | 0.14 (-0.13 ~ 0.40) | 0.319 |
| Hyperactivity Index | 0.24 (0.08 ~ 0.41) | 0.005 | 0.34 (0.08 ~ 0.59) | 0.015 | 0.17 (-0.07 ~ 0.40) | 0.168 |
| **SRS Score** | | | | | | |
| Social Awareness | 1.18 (0.14 ~ 2.22) | 0.028 | 0.93 (-0.61 ~ 2.47) | 0.243 | 1.57 (-0.02 ~ 3.16) | 0.060 |
| Social Cognition | 1.13 (-0.32 ~ 2.58) | 0.129 | 1.31 (-1.03 ~ 3.65) | 0.281 | 0.70 (-1.31 ~ 2.71) | 0.501 |
| Social Communication | 3.25 (0.87 ~ 5.62) | 0.009 | 3.04 (-0.21 ~ 6.29) | 0.054 | 2.71 (-0.95 ~ 6.37) | 0.154 |
| Social Motivation | 1.15 (-0.25 ~ 2.56) | 0.110 | 1.15 (-0.97 ~ 3.27) | 0.295 | 1.38 (-0.71 ~ 3.47) | 0.204 |
| Autistic Mannerisms | 0.86 (-0.77 ~ 2.49) | 0.305 | 0.06 (-2.20 ~ 2.33) | 0.957 | 0.72 (-1.80 ~ 3.24) | 0.579 |
| Total Score | 7.57 (1.70 ~ 13.44) | 0.013 | 6.99 (-1.46 ~ 15.44) | 0.113 | 7.08 (-1.62 ~ 15.77) | 0.118 |
| **Total model:** adjusted for gestational age at birth, child sex, maternal abnormal pregnancy history, first-trimester BMI, household income, and parental education; **Sex-stratified models (boys and girls):** adjusted for gestational age at birth, maternal abnormal pregnancy history, first-trimester BMI, household income, and parental education  **Abbreviations:** IH, isolated hypothyroxinemia; CDSC-II, Chinese developmental scale for children aged 0-6 Years (2^nd^ Edition); DQ, development quotient; CPSQ, Conners parent symptom questionnaire; SRS, social responsiveness scale; CI, Confidence Interval | | | | | | |
